# Analysis of Trace Elements in Methamphetamine Hydrochloride by Inductively Coupled Plasma-Mass Spectrometry

**DOI:** 10.6028/jres.093.122

**Published:** 1988-06-01

**Authors:** Tohru Kishi

**Affiliations:** National Research Institute of Police Science 6, Sanbancho, Chiyoda-ku Tokyo 102, Japan

## Introduction

Drug abuse is a serious problem in the world and these drugs are synthesized in clandestine laboratories. The determination of the manufacturing source provides important information regarding drug traffic [1,2]. One of the stimulants imported illegally, methamphetamine, is a serious social problem in Japan.

**Figure 1:**
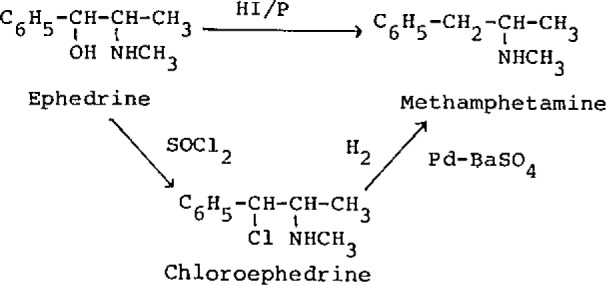
Synthesis of methamphetamine from ephedrine.

Methamphetamine is synthesized from ephedrine or methyl benzyl ketone as the raw material. In Asian areas, ephedrine is used as a starting material and methamphetamine is prepared by catalytic reduction of chloroephedrine by palladium-barium sulfate [3] or by reduction of ephedrine with hydrogen iodide and red phosphorus [4] as shown in figure 1.

The identification of catalyst and/or reagents based on final product analysis have been studied by radiochemical neutron activation analysis (RNAA) [2]. Palladium, barium, and iodine were determined as trace elements in methamphetamine hydrochloride.

In this paper, these trace elements were determined directly by inductively coupled plasma-mass spectrometry (ICP-MS) and the results are compared with those of neutron activation analysis (NAA).

## Experimental

### Reagents

Methamphetamine hydrochloride was prepared by Emde’s method [3] or Nagai’s method [4] as shown in figure 1.

### Procedure

For the ICP-MS, samples were dissolved in water in a concentration of 0.1–1%, and then the analytes determined by the Seiko ICP-MS SPQ-6100. The plasma operating conditions were: rf power 1.35 kW; nebulizer gas flow rate, 0.5 L/min; plasma gas rate, 16 L/min; auxiliary gas rate, 1 L/min. The plasma was sampled at a depth of 8 mm from the load coil. The mass spectrometer was a quadrupole type, and the measurement time was 2 ms per point with a scan increment of 0.125 amu. For quantitative analysis, the intensity of Na-23, Br-79, Pd-106, I-127 and Ba-138 were integrated.

For NAA, samples were sealed in polyethylene bags. After 5 hours irradiation in a thermal neutron flux (1 × 10 n·cm^−2^·s^−1^), Na-24 and Br-82 were directly determined while Pd-109 and Ba-131 were precipitated as palladium-dimethylglyoxime complex and barium sulfate, respectively. After 5 min irradiation, I-128 was determined in the chloroform layer after radiochemical separation.

## Results and Discussion

### Mass Spectra of Methamphetamine Hydrochloride

**Figure 2:**
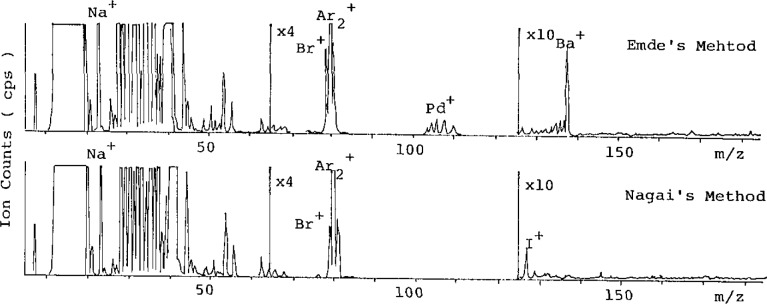
ICP-MS spectra of methamphetamine hydrochloride.

ICP/MS spectra of methamphetamine hydrochloride solution are shown in figure 2 for the mass range *m/z* 1 to 200. Barium, palladium, bromine, and sodium, were detected in the methamphetamine hydrochloride prepared by Emde’s method and iodine, bromine, and sodium were detected in the methamphetamine hydrochloride prepared by Nagai’s method. Barium and palladium originated from palladium-barium sulfate catalyst and sodium and bromine from sodium hydroxide and hydrochloric acid as neutralization reagents. Iodine originated from hydrogen iodide.

For the mass range *m/z* 20 to 70, many ions were observed, because the sample solution contained large amounts of nitrogen and chlorine; thus, further investigation is required for the determination of trace elements in this region.

### Analytical Calibration Curve

**Figure 3:**
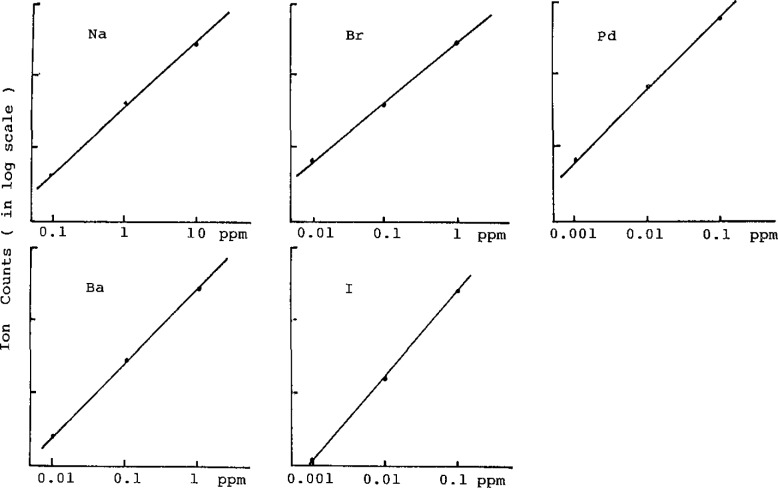
Analytical calibration curve of Na, Br, Pd, Ba, and I.

The analytical calibration curves shown in figure 3, obtained in the integration mode, show a useful working range of 3 orders of magnitude. These were linear for 0.1–10 μg/mL of sodium, for 0.01–1 μg/L of bromine and iodine and for 0.001–0.1 μg/mL of palladium and barium.

These data were obtained from reference solutions containing palladium, barium, iodine, bromine and sodium. The range of these calibration curves was established by considering the practical concentration of each element [2]. Therefore these elements were determined simultaneously, when one sample solution was measured.

### Quantitative Analysis

Trace elemental concentrations determined by ICP-MS are listed in table 1. The result of INAA and RNAA are listed in parentheses. The results of ICP-MS and NAA are almost the same except for concentrations of bromine. Concentrations of bromine by ICP-MS were larger than those by NAA. Argon dimer ion peak (*m/z* 80) is very close to the bromine ion peak (*m/z* 79). Consequently, it might interfere in the peak area calculation of bromine peak (*m/z* 79).

## Conclusion

The trace elements in methamphetamine hydrochloride (stimulant) were determined by ICP- MS and NAA. Part per million levels of sodium, bromine, palladium, barium and iodine, which originated from the catalyst and/or reagents, were found in the methamphetamine hydrochloride crystal. From these results, trace amounts of reagent and catalyst remained in the final product and a specific synthetic method could be determined by analysis of the methamphetamine prepared in clandestine laboratories.

## Figures and Tables

**Table 1:** Trace elements in methamphetamine hydrochloride by ICP-MS (μg/g)

Synthetic method	Na	Br	Pd	I	Ba
Emde’s	240(220)	130(80)	1.0(1.0)	–	1.0(1.0)
Nagai’s	20(20)	90(80)	–	5(7)	–

(): Determined by INAA and RNAA

– : Not detected
